# Gallbladder Neuroendocrine Carcinoma: A Rare Endocrine Tumor

**DOI:** 10.7759/cureus.7487

**Published:** 2020-03-31

**Authors:** Suman Siddamreddy, Sreenath Meegada, Anum Syed, Mujtaba Sarwar, Vijayadershan Muppidi

**Affiliations:** 1 Internal Medicine, Baptist Health Medical Center, Little Rock, USA; 2 Internal Medicine, The University of Texas Health Science Center/Christus Good Shepherd Medical Center, Longview, USA; 3 Internal Medicine, St. Vincent Health System, Little Rock, USA; 4 Internal Medicine, Indiana University Health, Indianapolis, USA

**Keywords:** gallbladder, neuroendocrine tumor, neuroendocrine carcinoma, gallbladder neuroendrocrine carcinoma

## Abstract

Gallbladder neuroendocrine neoplasms (GB-NEN) are very rare neuroendocrine tumors (NETs). GB-NEN can present as carcinoid or typical/atypical carcinoid or small cell carcinoma. Most of the GB-NENs present as gall bladder polyps or stones with right upper quadrant pain, nausea and non-specific symptoms which leads to clinical misdiagnosis. Considering the rare occurrence of GB-NENs, and lack of multi-center research data there is no unified standard for identification and treatment. We here present an 84-year-old male presenting with right upper quadrant and epigastric pain, and eventually diagnosed with mixed cell (more of small cells mixed with intermediate to large cells) neuroendocrine cancer of gall bladder.

## Introduction

Neuroendocrine tumors (NETs) are a group of neoplasms that originate from neuroendocrine cells present in the various organs but more commonly found in gastrointestinal (GI) tract, lungs, and thyroid [1]. In the GI tract, they are more common in ileum, jejunum, and pancreas, but very rarely reported in gallbladder as well [2]. As it is very rare aggressive neoplasm, we need more research to identify it earlier and develop a standardized treatment option.

## Case presentation

An 84-year-old Caucasian male presented to the emergency room with one day history of abdominal pain, more localized to epigastric and right upper quadrant area. Pain is sharp in intensity, radiating to substernal area. Pain was associated with few episodes of nausea and vomiting. He denied any hematemesis, diarrhea, shortness of breath, wheezing, fever and chills. He never had any similar complaints in the past. He denied any weight loss or loss of appetite. His pain slightly got better in the emergency room with intravenous opiates. His past medical history was significant for essential hypertension, Alzheimer’s dementia, coronary artery disease, atrial fibrillation and stroke. Past surgical history was significant for appendectomy, coronary artery by-pass grafting and joint surgeries.

His initial vitals were within normal limits. On physical exam he had irregularly irregular rhythm with good breath sounds bilaterally. Abdomen examination was significant for right upper quadrant tenderness with positive murphy sign (tenderness and guarding in the right upper quadrant of abdomen on palpation and exacerbated by inspiration).

Significant initial laboratory findings on initial presentation include elevated alkaline phosphate 273 IU/ml (reference range: 40-150 IU/ml), elevated aspartate aminotransferase (AST) -63 IU/ml (reference range: 5-34 IU/ml), elevated total bilirubin of 1.4 mg/dl (reference range: 0.2-1.2 mg/dl), mildly elevated lipase of 101 IU/ml (reference range: 8-78 IU/ml), normal alanine transaminase (ALT) 44 IU/ml (reference range: 0-55 IU/ml), and normal white blood cell count 5.9 k/cmm (reference range: 5-10 k/cmm).

**Figure 1:**
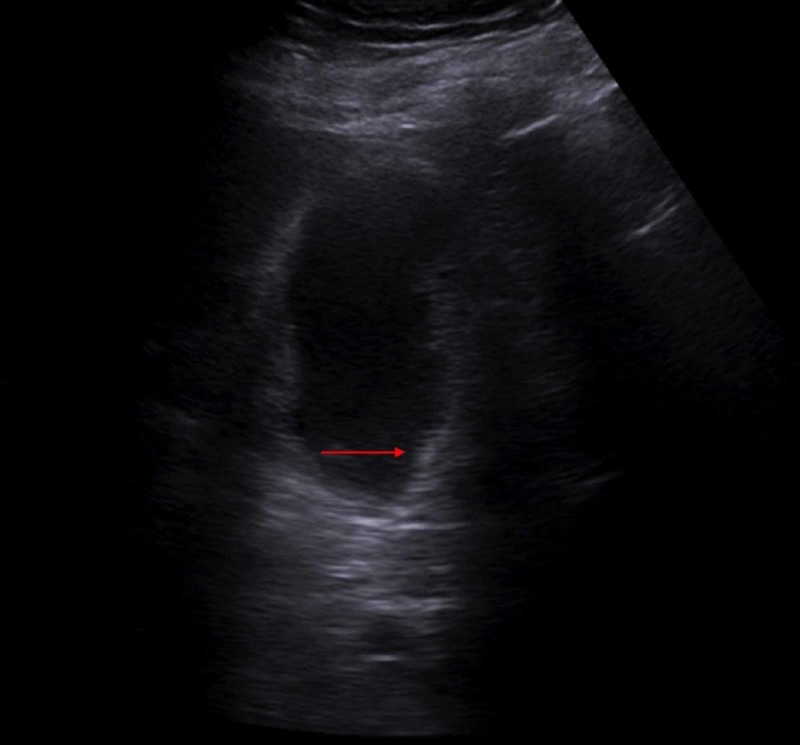
Ultrasound showing inspissated gall bladder (arrow pointing)

**Figure 2:**
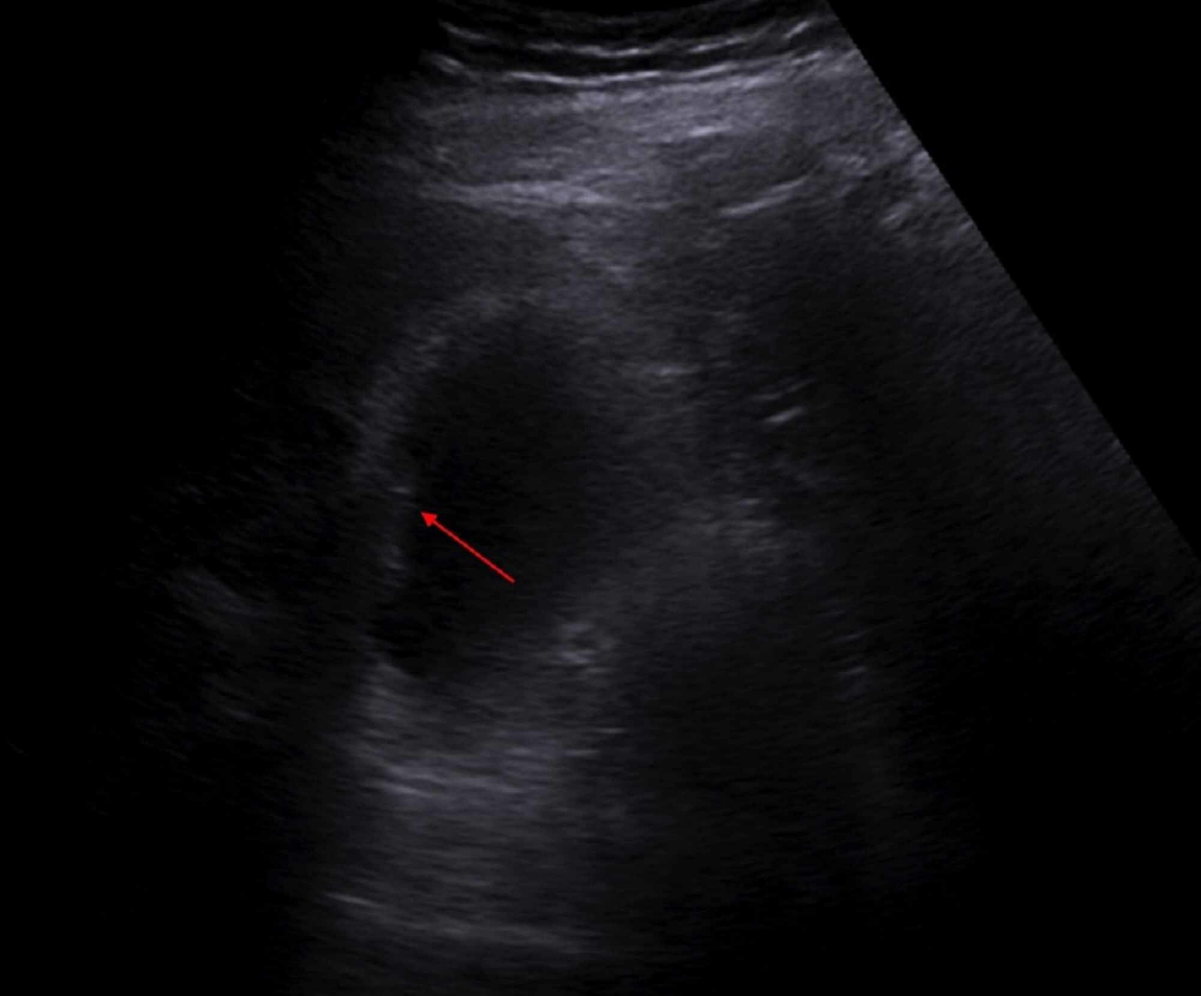
Ultrasound gall bladder showing non-specific, no dependent tissue along the wall (arrow pointing)

Because of clinical suspicion of acute cholecystitis, the patient had an ultrasound of gallbladder which showed cholelithiasis, and sludge/inspissated gall bladder (Figure 1) with no radiographic evidence of acute cholecystitis, non-specific, soft, non-dependent tissue along the gall bladder wall (Figure 2).

The patient was started on intravenous Levofloxacin and admitted to the hospital with a surgical consultation.

The patient had a laparoscopic cholecystectomy with normal intra-operative cholangiogram during that hospitalization. He was found to have a gangrenous gallbladder with a large Calot node (sentinel lymph node of gall bladder) and nodule on the wall of gallbladder and it was sent for biopsy after removal. He had an uneventful postoperative state and was discharged home the next day.

Biopsy results showed high-grade neuroendocrine carcinoma of gallbladder with predominantly small cell cancer. It was poorly differentiated, invading lymphovascular structures, perimuscular fat and serosa. Unfortunately, lymph nodes were not sampled during the surgery as the suspicion for malignancy was low.

**Figure 3:**
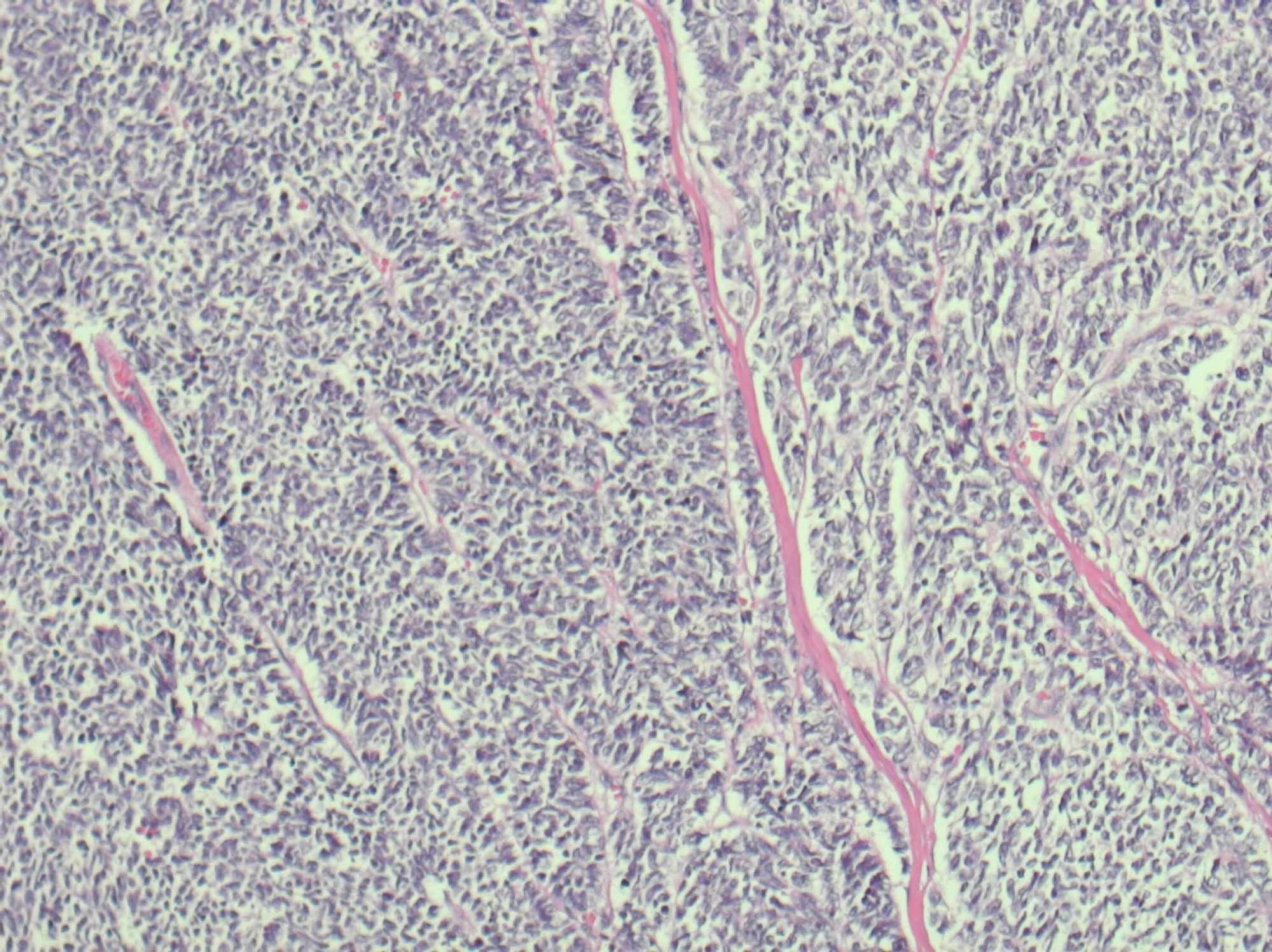
Histological examination of tumor showing mostly small cells mixed with intermediate to large cells (Blue dotted cells) with hyperchromatic nuclei and scant cytoplasm

**Figure 4:**
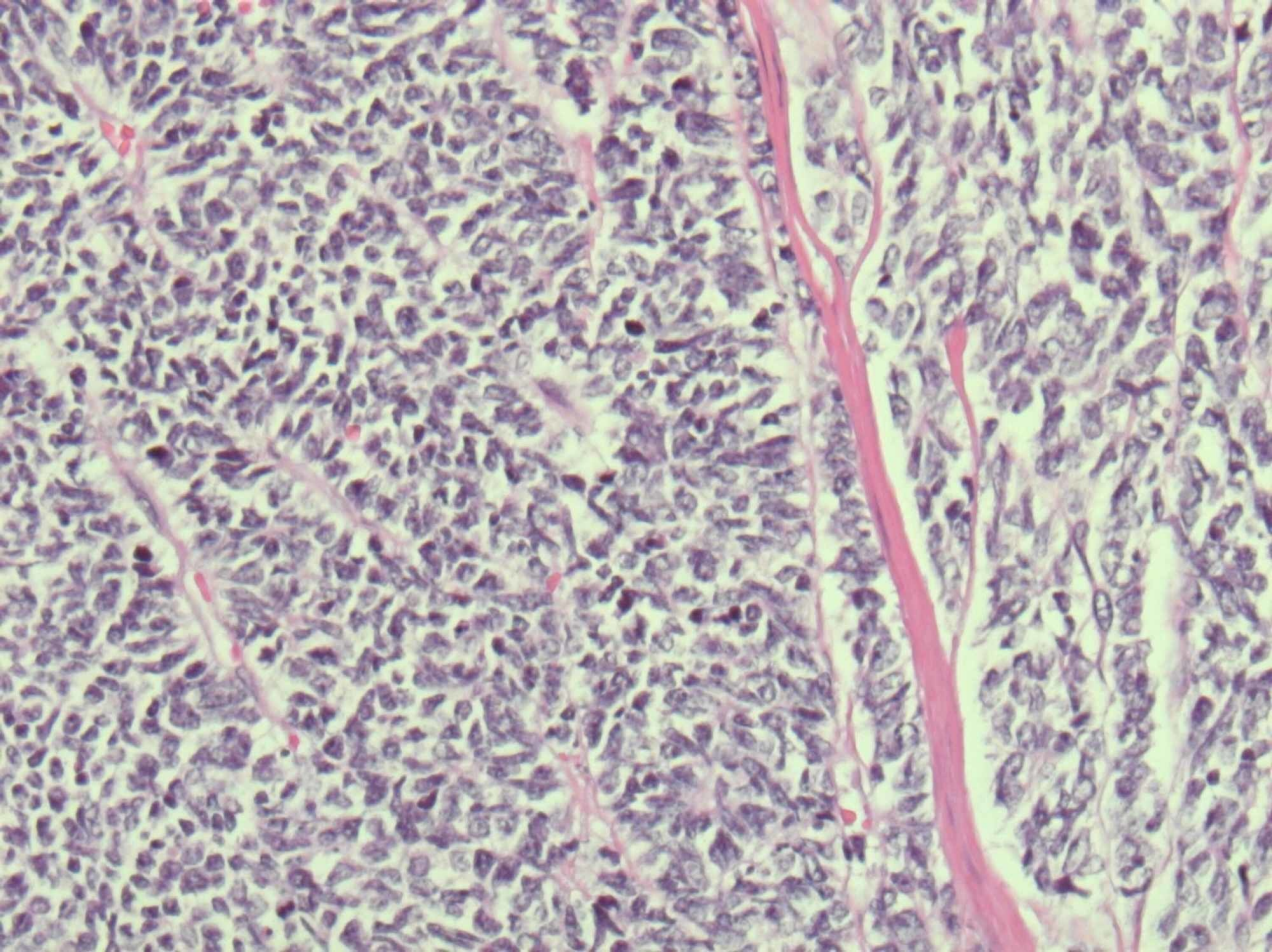
Histological examination of tumor showing mostly small cells mixed with intermediate to large cells (Blue dotted cells) with hyperchromatic nuclei and scant cytoplasm (Magnified slide) with lymphocytic invasion (pink colored lymph vessel with blue dot cells)

Pathological staging showed pT3, pNx. Sections showed abundant neuroendocrine tumor with associated necrosis. Most of the cells were small in size with few intermediate to large cells (Figures 3, 4).

Immunohistochemistry reveals that malignant cells are positive for pan keratin, low molecular weight keratin, synaptophysin, TTF1, CK7 and CA19-9. Cells are negative for CD20, CD3, CK20 and p63.

His staging studies including computerized tomography (CT) scanning of chest, abdomen, pelvis, head, and nuclear medicine whole body bone scan were negative for any metastatic lesions. Oncologist diagnosed him with Stage IIIA (pT3, Nx Mx) small cell carcinoma of the gallbladder and arranged for adjuvant chemoradiation with Capecitabine as radio sensitizing agent. The patient was supposed to be started on a small cell cancer chemotherapy regimen, however he decompensated in the next few days and died after family has opted for comfort care measures only.

## Discussion

Neuroendocrine neoplasms (NEN) are a group of neoplasms that can arise in various organs such as gastrointestinal tract, lungs, thyroid that usually have predominate neuroendocrine differentiation. Incidence of neuroendocrine tumors is about 5.25/100,000 [3]. Gallbladder NETs only comprise 5% of those [4]. There are no clear classification systems as some studies classify based on organ where they arise from, and some classify based on histological grading and staging. International Agency for Research on cancer (IARC) and World Health Organization (WHO) experts proposed a classification system for NENs in 2018 [5]. Most of the physicians use a classification system endorsed for gastroenteropancreatic (GEP) system NETs by WHO [6]. WHO classification is mainly based on the tumor proliferative rate as assessed using mitotic counts or Ki-67 labelling index. Basically, there are two main categories of GEP NENs: Well-differentiated neuroendocrine tumors (NETs) and poorly differentiated neuroendocrine carcinomas (NECs).

Morphology

1) Well-differentiated NETs are well circumscribed with relatively uniform cells and nuclei with granular cytoplasm and stippled chromatin. Most of them secrete neuro-secretory granules and express markers such as synaptophysin and chromogranin A. There are some tumors that specifically secrete hormones such as insulin, glucagon, gastrin, somatostatin, etc. Most of the time, we cannot correlate the histological features with morphological location except for few specific NETs like insulin secreting pancreatic NETs.

2) Poorly-differentiated NECs on the other hand have non-uniform cells with irregular nuclei and less cytoplasmic granularity. They can still have some immune reactivity but not as much as NETs. There is no definitive theory on origin for gallbladder NECs. Some researchers think that gallbladder epithelium becomes metaplastic and changes to neuroendocrine cells because of chronic inflammation in patients with cholelithiasis, while some others think that NEC is transformed from gallbladder adenocarcinoma [7,8].

Clinical features and diagnosis

There are no specific symptoms and signs that are characteristic for gallbladder NECs. Patients most commonly present with right upper quadrant pain, nausea and vomiting, and positive Murphy's sign. Some patients present with weight loss and loss of appetite. Very rarely, patients present with symptoms of carcinoid syndrome including flushing, edema, and wheezing. Liver function tests, complete blood counts, basic metabolic panel are usually done in these patients on presentation. Imaging studies like ultrasonography, CT or magnetic resonance imaging (MRI) help in identifying the lesions. Most of the times we cannot distinguish them from other types of gallbladder cancers just based on imaging studies. A definitive diagnosis of gallbladder NECs is made only based on pathology reports and immunohistochemistry staining. It also helps with tumor grading and staging based on WHO classification. Patients also need other tests like bone scans, positron emission tomography (PET) scans, serum tumor markers, etc. to look for metastatic lesions that will help in staging and eventually with treatment.

Treatment

The treatment options for gallbladder NEC vary based on the stage of tumors. Early in situ tumors usually respond well to cholecystectomy alone [9]. But late stage tumors need radical surgeries along with dissection of nodes and even removal of some close metastatic lesions [10]. As most of these patients present in advanced stages, they need concomitant chemoradiation. Despite all above treatment options, gallbladder NEC still has very high mortality with poor prognosis.

## Conclusions

Neuroendocrine carcinoma of gallbladder is an exceedingly rare malignancy with poor prognosis. The poor prognosis, in part, is based on the vague symptoms on presentation and advanced stage of presentation. Because of the rarity of the disease, we need more research to diagnose these patients early and come up with a standardized treatment plan.
